# Correlation Between APOBEC3B Expression and Clinical Characterization in Lower-Grade Gliomas

**DOI:** 10.3389/fonc.2021.625838

**Published:** 2021-03-26

**Authors:** Hao Zhang, Zhiyang Chen, Zeyu Wang, Ziyu Dai, Zhengang Hu, Xun Zhang, Min Hu, Zhixiong Liu, Quan Cheng

**Affiliations:** ^1^ Department of Neurosurgery, Xiangya Hospital, Central South University, Changsha, China; ^2^ Department of Laboratory Medicine, The Second Xiangya Hospital, Central South University, Changsha, China; ^3^ Department of Clinical Pharmacology, Xiangya Hospital, Central South University, Changsha, China

**Keywords:** glioma, APOBEC3B, tumor microenvironment, immune response, prognosis

## Abstract

**Background:**

As the most aggressive tumors in the central nervous system, gliomas have poor prognosis and limited therapy methods. Immunotherapy has become promising in the treatment of gliomas. Here, we explored the expression pattern of APOBEC3B, a genomic mutation inducer, in gliomas to assess its value as an immune biomarker and immunotherapeutic target.

**Methods:**

We mined transcriptional data from two publicly available genomic datasets, TCGA and CGGA, to investigate the relevance between APOBEC3B and clinical characterizations including tumor classifications, patient prognosis, and immune infiltrating features in gliomas. We especially explored the correlation between APOBEC3B and tumor mutations. Samples from Xiangya cohort were used for immunohistochemistry staining.

**Results:**

Our findings demonstrated that APOBEC3B expression level was relatively high in advanced gliomas and other cancer types, which indicated poorer prognosis. APOBEC3B also stratified patients’ survival in Xiangya cohort. APOBEC3B was significantly associated with infiltrating immune and stromal cell types in the tumor microenvironment. Notably, APOBEC3B was involved in tumor mutation and strongly correlated with the regulation of oncogenic genes.

**Conclusion:**

Our findings identified that APOBEC3B could be a latent molecular target in gliomas.

## Introduction

Diffuse gliomas, including lower-grade gliomas (LGGs) and glioblastomas (GBMs), are the most malignant brain tumor in adults (1). Glioma patients always had high mortality rate, high recurrence risk and dismal prognosis (2). LGGs comprised of diffuse low-grade and intermediated-grade gliomas. Nowadays, the primary therapeutic methods for LGG is surgery with concurrent radiotherapy and chemotherapy (3). Despite the advances in treatment methods, the median overall survival of LGG patients is less than 2 years due to limitations in therapeutic options. Therefore, novel therapeutic strategies are urgently needed. In recent years, immunotherapy, including immune checkpoint inhibitors, has demonstrated remarkable results in cancer treatment and casted new lights on clinical management of glioma (4, 5).

The tumor microenvironment (TME) is a highly dynamic composition of various cell types and is considered being responsible for the effectiveness of immunotherapies. An immunosuppressive TME was formed during the progression and recurrence of glioma (6). Immune infiltrating cells account for the major part of TME and sometimes can protect tumor cells from being detected and exterminated by the immune system. For example, regulatory T cells (Tregs) and tumor-associated macrophages (TAMs) have been proved to exert immunosuppressive effect in glioma (7). As another important member in the TME, immune checkpoint molecules are also involved in immunosuppressive mechanism. Immunotherapy of immune checkpoint blockade has become a promising treatment modality for cancers (8).

APOBEC3B, a member of APOBEC (apolipoprotein B mRNA editing enzyme, catalytic-polypeptide-like) enzymes with cytidine deaminase activity (9), can induce prevalent mutagen of genomic DNA in multiple cancers. APOBEC3B has been found to be upregulated in various cancer types with poor prognosis (10–12), and is also considered as a mediator regulating the growth, the metastatic outgrowths, and the emerging therapeutic resistance of cancer cells (13). High expression of APOBEC3B is associated with immune evasion of cancer (14). Notably, high expression of APOBEC3B also enhances the sensitivity to immune checkpoint blockade in melanoma (15). However, the relationship between TME and the APOBEC3B expression in gliomas remains largely unknown.

Therefore, we integrated and analyzed the RNA-sequencing data of glioma patients from The Cancer Genome Atlas (TCGA) and Chinese Glioma Genome Atlas (CGGA) databases to reveal the immune features and clinical characteristics of APOBEC3B in gliomas.

## Methods

### Data Collection

This study was ethically approved by Xiangya Hospital, Central South University. Archived paraffin embedded glioma tissues (WHO grades II–IV) were collected from patients (n = 58) who underwent surgery in the Department of Neurosurgery, Xiangya Hospital, Central South University. We collected transcriptomic data of LGG and GBM samples from the TCGA and CGGA datasets, and RNA seq was used for the analysis. RNA-seq data about specific tumor anatomic structure in GBM was downloaded from Ivy Glioblastoma Atlas Project (http://glioblastoma.alleninstitute.org/). APOBEC3B expression data in distinct radiographical areas of normal brain and GBM was downloaded from the Gill dataset.

### Immunohistochemistry

Tissues of different grades of human gliomas (WHO grades II–IV) were formalin-fixed and paraffin-embedded to obtain sections (4 mm). Sections were then boiled in sodium citrate buffer (pH 6.0) for antigen retrieval, 3% H_2_O_2_ was used for blockage of endogenous HRP activity. Slides were blocked with 10% normal goat serum and incubated with primary antibody (rabbit polyclonal anti-APOBEC3B antibody, 1:50; Proteintech; Wuhan, China) at 4°C overnight. Signal was visualized with horse radish peroxidase conjugated secondary antibody and 3, 3′-diaminobenzidine (DAB) as the substrate. Slides were counterstained with hematoxylin, and representative images were obtained using an Olympus inverted microscope. H-score of glioma samples was subsequently calculated.

### Bioinformatic Analysis

We acquired the chromosome localization of APOBEC3B on the GeneCards database (https://www.genecards.org/). APOBEC3B gene structure was analyzed on the Ensembl database (http://asia.ensembl.org/), with its protein structure analyzed in the Uniprot database (http://www.uniprot.org/). APOBEC3B gene structure was then visualized by using Illustrator for biological sequences software (IBS, http://ibs.biocuckoo.org/). The protein sequence comparison among different species was analyzed by DNAMAN software (lynnonBiosoft, USA). Correlation analysis of APOBEC3B was performed using gene expression profiles from the TCGA and CGGA datasets with R language (https://www.r-project.org/). Somatic mutations and somatic copy number alternations (CNAs) of the cases with the corresponding RNA-seq data were downloaded from TCGA database. GSITIC analysis was adopted to determine the genomic event enrichment. CNAs associated with APOBEC3B expression and the threshold copy number (CN) at alteration peaks were from GISTIC 2.0 analysis (https://gatkforums.broadinstitute.org). GSITIC analysis was performed based on the first 25% and last 25% of samples. The gene sets variation analysis (GSVA) package was used to analyze the differential expression in GO terms of immune related process and immune cell lineages from TCGA and CGGA. As for somatic mutations, software VarScan2 was used to detect WES data of APOBEC3B^high^ and APOBEC3B^low^ groups. P <0.05 was set as the criteria for selecting differentially mutated genes, and Fisher’s exact test was used to identify the differentially mutation pattern. CoMEt algorithm was used to detect the co-occurrence and mutually exclusive mutations. R package maftools was used for the visualization of the somatic mutations. Correlation analysis was performed by the expression values of APOBEC3B and GO term, and the items with p <0.05 and high correlation coefficient were selected. After Spearman correlation analysis, Heatmap was used to construct gene ontology (GO) analysis of the most correlated genes. The relevant immune signaling pathways of high level of APOBEC3B expression from GO were analyzed by ClueGO (16). ClueGO: a Cytoscape plug-in to decipher functionally grouped gene ontology and pathway annotation networks. ESTIMATE (Estimation of Stromal and Immune cells in Malignant Tumor tissues using Expression) algorithm was used to evaluate the infiltration of immune cells and the presence of stromal cells in tumor samples, which generated three results including immune score (reflecting the level of immune cells infiltrations in tumor tissue), stromal score (reflecting the presence of stroma in tumor tissue), and estimate score (reflecting tumor purity).

We analyzed the relationship between APOBEC3B expression and overall survival (OS) in adrenocortical carcinoma (ACC), cholangiocarcinoma (CHOL), esophageal carcinoma (ESCA), liver hepatocellular carcinoma (LIHC), lung adenocarcinoma (LUAD), pancreatic adenocarcinoma (PAAD), Uterine Corpus Endometrial Carcinoma (UCEC), Uterine Carcinosarcoma (UCS), and Kidney Chromophobe (KICH) cancer types based on the pan-cancer data in TCGA dataset. We also analyzed the correlation between APOBEC3B expression and the abundance of six immune infiltrating cell types, including activated CD4^+^T cell, central memory CD8^+^T cell, macrophage, Myeloid-derived suppressor cells (MDSCs), memory B cell and type 2 helper cell in pan-cancer from TCGA.

The weighted gene co-expression network analysis (WGCNA) package in R was used to perform WGCNA. The expression profile of 2,559 APOBEC3B related genes (correlation efficient >0.4) was applied as the input of WGCNA. The association between individual genes and APOBEC3B density was quantified by gene significance, and the correlation between module eigengenes and gene expression profiles was represented by module membership. A power of *β* = 3 and a scale-free R2 = 0.87 were set as soft-threshold parameters to ensure a scale-free topology network. A total of eight modules were generated, and yellow module showed the highest correlation (r = 0.96, p = 1.6e-112). Genes within the yellow module were chosen for further GO and KEGG enrichment analysis.

### Statistical Analysis

Spearman correlation analysis was used to evaluate the correlations between continuous variables. The survival probability was described by Kaplan–Meier survival curves. Patients were stratified according to the median value of APOBEC3B or the cutpoint value automatically calculated. The Student t-test was used to determine the expression levels of APOBEC3B with regard to pathological characteristics. The linear relationship between gene expression levels was evaluated by the Pearson correlation. All statistical analyses were performed using R project (version 3.6.1, https://www.r-project.org/). P-values <0.05 were considered to be statistically significant. And all tests were two-sided.

## Results

### The Expression Level of APOBEC3B Is Increased in Aggressive Glioma and Other Cancers

**Figure 1:**
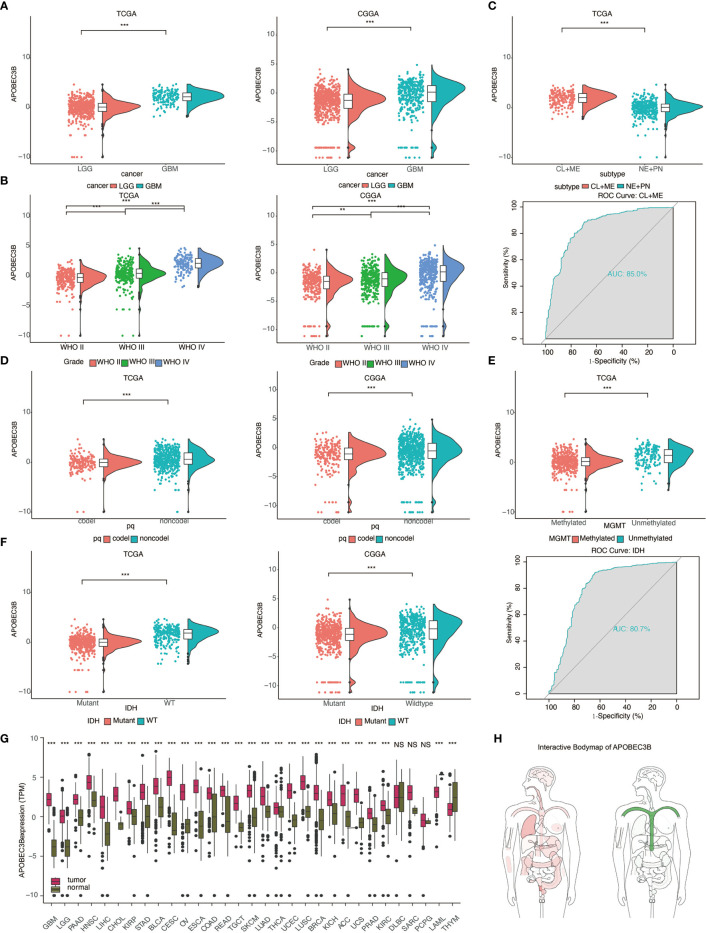
The expression level of APOBEC3B is increased in aggressive glioma and other cancers. Analysis of APOBEC3B mRNA levels in **(A)** LGG and GBM **(B)** WHO grade II–IV gliomas **(D)** 1p19q codeletion and non-codeletion from TCGA and CGGA datasets. **(C)** APOBEC3B expression in distinct subclasses(upper), ROC curve indicating sensitivity and specificity of APOBEC3B expression as a discriminative biomarker for CL+ME subtypes and GBM (lower). **(E)** APOBEC3B expression in MGMT methylated and unmethylated. **(F)** Analysis of APOBEC3B level in IDH mutant and wildtype from TCGA and CGGA. ROC curve indicates the sensitivity and specificity of APOBEC3B expression as a diagnostic biomarker for discriminate IDH mutation from non-IDH mutation. **(G)** APOBEC3B mRNA expression levels in pan-cancer. **(H)** The median expression of tumor (red) and normal (green) samples in bodymap. NS, Not Statistically Significant; *P < 0.05; **P < 0.01; ***P < 0.001; ****P < 0.0001.

The mRNA expression levels of APOBEC3B were measured using data from publicly accessed databases including over 1,600 gliomas samples: TCGA. n = 672: CGGA, n = 1013. We found that APOBEC3B was upregulated in GBM compared to LGG (P <.05, respectively; **Figure 1A**). The expression level of APOBEC3B was also increased in order of grade II, grade III, and grade IV (WHO classification) (P <.05,respectively; **Figure 1B**). Based on gene expression profiling, glioblastoma can be classified into four distinct molecular subtypes: classical (CL), mesenchymal (ME), proneural (PN) and neural (NE). Practically, the CL and ME types predict worse clinical prognosis. To figure out the relationship between APOBEC3B and molecular subtypes, we further investigated the expression level of APOBEC3B among subtypes: increased expression level of APBOEC3B was found in CL and ME compared to PN and NE (P <.05, respectively; **Figure 1C**). ROC further indicated that APOBEC3B expression level can distinguish CL and ME from GBM (area under curve (AUC) value = 0.85; P < 0.05; **Figure 1C**).

We also examined the relationship between APOBEC3B and certain genomic alterations. Glioma patients with codeletion of 1p and 19q derived more benefits in several clinical trials (17). We observed that the expression of APOBEC3B was decreased in the 1p19q codeletion cluster in pan-glioma analysis (P <.05, respectively; **Figure 1D**). Better clinical outcome was accompanied by MGMT promoter methylated subtype (18) and IDH*mut* (19), similarly, down-regulated of APOBEC3B mRNA expression level was found in these two types compared to wild type patients (**Figures 1E, F**), and receiver operating characteristic (ROC) curve analysis indicated that the expression of APOBEC3B discriminated IDH mutation from non-IDH mutation in pan-glioma analysis (the area under the curve (AUC) value = 0.807; P < 0.05; **Figure 1F**). Among nine methylation probes designed for APOBEC3B from TCGA, all of them exhibited remarkable negative association with expression of APOBEC3B, which most of the association was statistically significant (**Figure S1**).

Furthermore, we analyzed various clinically related characteristics of APOBEC3B in gliomas. Pathologically, APOBEC3B has been found to be most adequately expressed in microvascular proliferation (MVP) (**Figure S2A**). In copy number (CN) analysis, glioma with APOBEC3B CN loss expressed higher level of APOBEC3B mRNA (**Figure S2B**). Radiographically, APOBEC3B was upregulated in contrast enhancing area compared with non-contrast enhancing and normal brain area (**Figure S2C**). Moreover, the expression pattern of APOBEC3B with regard to the histology of gliomas was shown in **Figure S2D**. We also examined APOBEC3B level in primary, recurrent, and secondary patients respectively; statistics revealed that APOBEC3B expression was higher in recurrent patients than in primary patients (**Figure S2E**). And in patients with different treatment outcomes, the expression of APOBEC3B was significantly higher in progressive patients than in patients who were in complete remission (**Figure S2F**).

APOBEC3B mRNA expression levels were analyzed in pan-cancer (**Figure 1G**) and interactive body map of APOBEC3B (**Figure 1H**). The results elucidated that besides LGG and GBM, expression of APOBEC3B was higher in multiple cancers including PAAD, Head and Neck Squamous Cell Carcinoma (HNSC), LIHC, CHOL, kidney renal papillary cell carcinoma (KIRP), stomach adenocarcinoma (STAD), Bladder Urothelial Carcinoma (BLCA), cervical squamous cell carcinoma and endocervical adenocarcinoma (CESC), ovarian cancer (OV), ESCA, colon adenocarcinoma (COAD), rectal adenocarcinoma (READ), Testicular Germ Cell Tumors (TGCT), Skin Cutaneous Melanoma (SKCM), LUAD, Thyroid Carcinoma (THCA), UCEC, lung squamous cell carcinoma (LUSC), Breast Invasive Carcinoma (BRCA), KICH, ACC, UCS, Prostate Adenocarcinoma (PRAD), kidney renal clear cell carcinoma (KIRC) than adjacent normal tissues, respectively, while lower in Thymoma (THYM).

### APOBEC3B Expression Is Elevated in Aggressive Glioma and Related to Poor Prognosis

**Figure 2:**
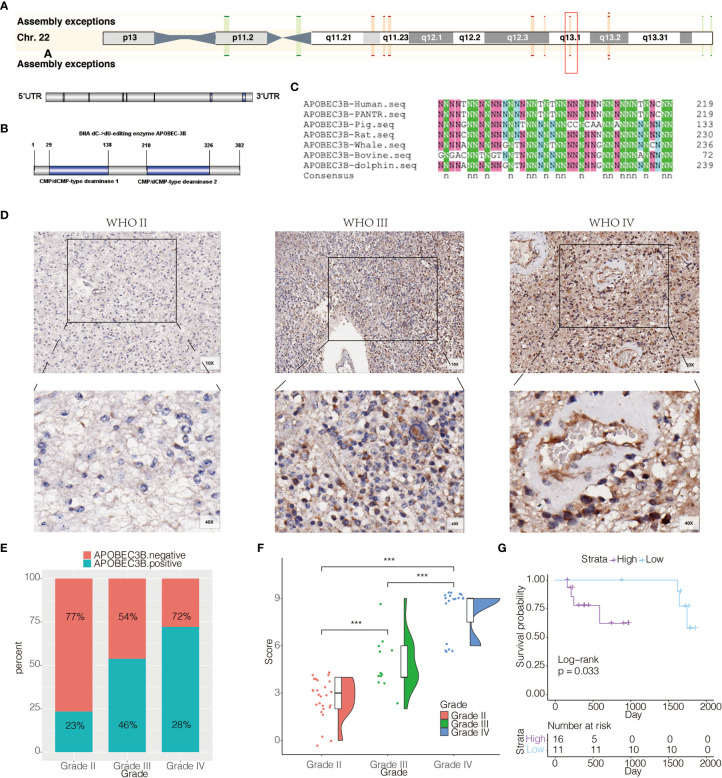
APOBEC3B expression is elevated in aggressive glioma and related to poor prognosis. **(A)** Chromosome localization and gene structure of APOBEC3B in human. **(B)** Structure of APOBEC3B. **(C)** Comparison of protein sequences encoded by APOBEC3B among seven different species. **(D)** Representative images of IHC staining for APOBEC3B in different pathological grades of gliomas [WHO II (27), WHO III (12), WHO IV (19)]. **(E)** Quantification of APOBEC3B IHC staining regarding the positive rate. **(F)** Quantification of APOBEC3B IHC staining regarding the H-score. **(G)** Overall survival based on high *vs* low expression of APOBEC3B in glioma patients (n = 27). The patients were stratified according to the H score of APOBEC3B in IHC staining. The H score has the range of 0–12. High group was defined as expression intensity >=6. Low group was defined as expression intensity <6. NS, Not Statistically Significant; *P < 0.05; **P < 0.01; ***P < 0.001; ****P < 0.0001.

APOBEC3B was located at 22q13.1 (**Figure 2A**), and the protein structure of APOBEC3B consisted of CMP/dCMP-type deaminase 1 and CMP/dCMP-type deaminase 2 (**Figure 2B**). In order to study the conservation of APOBEC3B among distinct species, we compared protein sequences encoded by APOBEC3B among seven different species (**Figure 2C**). Statistics showed that Homo sapiens APOBEC3B shared 75.71, 54.19, 78.73, 73.81, 44.76 and 74.52% identity to PANTR, pig, rat, whale, bovine, and dolphin, respectively. It presented that APOBEC3B was highly conserved in most kinds of mammals, but varied significantly between human and bovine. To confirm that APOBEC3B expression was also upregulated at the protein level, we performed IHC staining of APOBEC3B based on an independent cohort consisting of different pathological grades of glioma samples (n = 58) from our institution. APOBEC3B was located in the nucleus and cytoplasm, and increase in order of WHO classification (**Figure 2D**). The quantification of IHC staining was shown in **Figures 2E, F**, in which there was an increase of APOBEC3B expression as tumor grade increased. We further investigated the prognostic value of APOBEC3B in glioma based on 27 clinical samples with survival information, and patients with higher expression of APOBEC3B are more likely to have shorter overall survival (**Figure 2G**).

### Higher APOBEC3B Expression Is Related to Poor Survival in Glioma and Multiple Cancers

**Figure 3:**
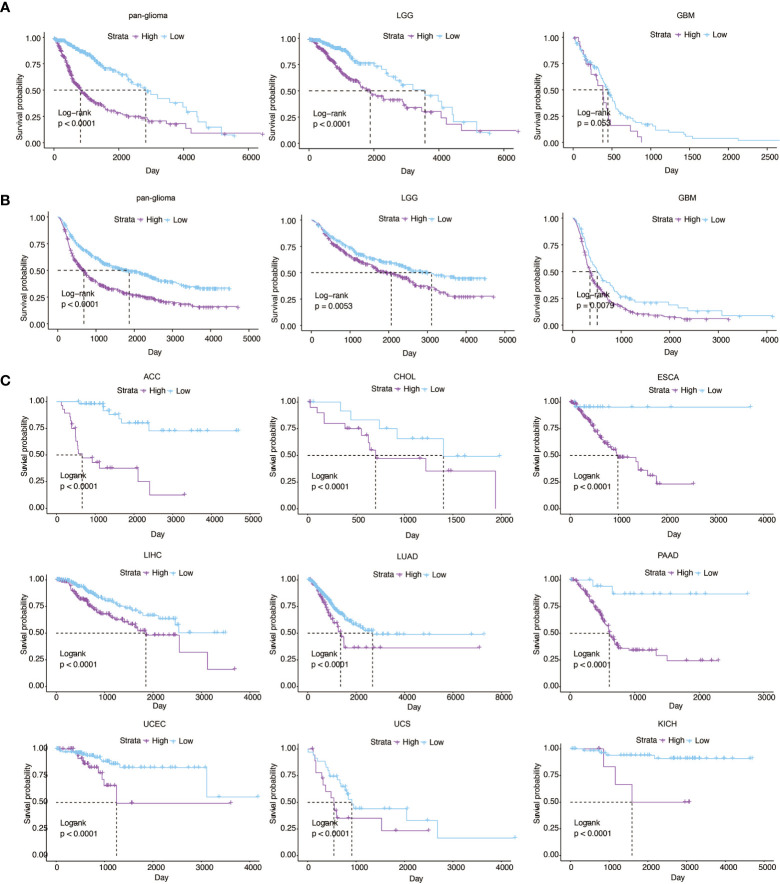
Higher APOBEC3B expression is related to poor survival in glioma and multiple cancers. Kaplan–Meier analysis of overall survival (OS) based on high *vs* low expression of APOBEC3B in pan-glioma analysis, LGG, and GBM patients in **(A)** TCGA and **(B)** CGGA datasets. GBM patients were stratified according to the cutpoint value automatically calculated in TCGA, and the cutpoint value was 3.1278. Kaplan–Meier analysis of overall survival (OS) based on high *vs* low expression of APOBEC3B in **(C)** ACC, CHOL, ESCA, LIHC, LUAD, PAAD, UCEC, UCS and KICH.

We used Kaplan–Meier analysis to subsequently explore the prognostic value of APOBEC3B in both TCGA and CGGA datasets. We revealed that APOBEC3B^high^ patients showed shorter overall survival (OS) than APOBEC3B^low^ patients in pan-glioma, LGG, and GBM (P <.05, respectively; **Figures 3A, B**). Thus, APOBEC3B might be a latent marker for prognosis in glioma patients. We further investigated the prognostic value of ABPOEC3B for pan-cancer, in which patients were divided into high and low APOBEC3B groups. High APOBEC3B expression was significantly correlated to worse prognosis in nine cancer types, including ACC, CHOL, ESCA, LIHC, LUAD, PAAD, UCEC, UCS, and KICH (P <.0001, respectively; **Figure 3C**).

### APOBEC3B Expression Is Related to Genomic Alterations in Glioma

**Figure 4:**
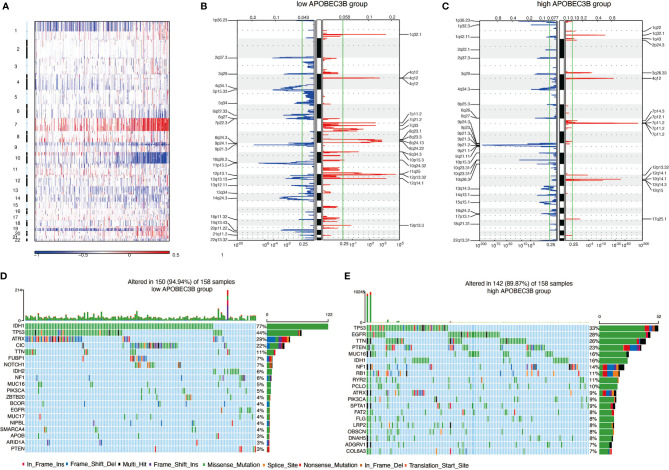
APOBEC3B expression is related to genomic alterations in glioma. **(A)** Overall CNV profile according to high *vs* low APOBEC3B expression. Blue (deletion); Red (amplification). Frequency of specific changes based on **(B)** APOBEC3B^low^ and **(C)** APOBEC3B^high^ groups. The X-axis represents the frequency of chromosomal deletion (blue) or amplification (red). Spectrum of somatic mutations in gliomas from **(D)** APOBEC3B^low^ and **(E)** APOBEC3B^high^ groups.

Genomic alterations can be easily found in glioma. Thus, we performed copy number variation (CNV) and somatic mutation analysis to examine whether there is a link between APOBEC3B expression levels and specific genomic alterations in glioma. An overall CNV profile comparison of APOBEC3B^high^(n = 158) and APOBEC3B^low^(n = 158) cluster was carried out. Besides the variation of chr1 and chr19, amplification of chr7 and deletion of chr10 most frequently occurred in glioma patients (**Figure 4A**). As a genomic symbol of oligodendroglioma, deletion of 1p and 19q tended to appear in APOBEC3B^low^ cluster (**Figure 4B**). Using GSITIC analysis, we found distinct genomic alterations in different clusters (**Figures 4B, C**). In APOBEC3B^low^ patients, PD-1 (2q37.3), CLPTM1L (5p15.33), CDKN2A (9p21.3), SAA1 (11p15.5) were frequently deleted, while HAS2 (8q24.13), NDRG1 (8q24.22), FGF23 (12p13.32) and CDK4 (12q14.1) were most frequently amplified. In APOBEC3B^high^ group, CDKN2A (9p21.3), PARK7 (1p36.23) and PTEN (10q23.31) were most frequently deleted, at the same time, EGFR (7p11.2) and CDK4(12q14.1) were most commonly amplified genes. Based on the level of APOBEC3B, somatic mutation profiles were analyzed. In low APOBEC3B group, IDH-1 (77%), TP53 (44%), ATRX (29%), and CIC (11%) are altered in high frequency, while TP53 (33%), EGFR (28%), TTN (26%), and PTEN (23%) were more frequently mutated in high APOBEC3B group (**Figures 4D, E**). Taken together, our results demonstrated that APOBEC3B expression level was pertinent to chromosomal alterations in glioma.

### Comparisons of Somatic Mutations Among Different Immune Infiltration Levels

**Figure 5:**
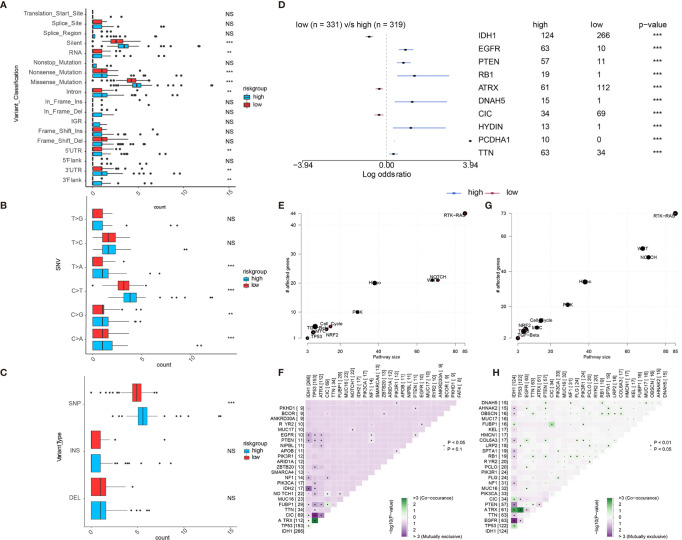
Landscape of somatic mutation in APOBEC3B^high^ and APOBEC3B^low^ groups. **(A–C)** the comparisons of mutation frequencies of **(A)** every mutation type classified by effects, **(B)** SNVs, **(C)** INDEL and SNP. **(D)** Forest plot shows the top 10 most significantly differentially mutated genes between two groups. Common carcinogenic pathways in **(E)** APOBEC3B^low^ and **(G)** APOBEC3N^high^ group. The heatmap indicates the mutually exclusive mutations and co-occurring of the frequently mutated genes in **(F)** APOBEC3B^low^ and **(H)** APOBEC3N^high^ groups. NS, Not Statistically Significant; *P < 0.05; **P < 0.01; ***P < 0.001; ****P < 0.0001.

We further used the R package maftools to analyze somatic mutations including the single-nucleotide variant (SNV), single-nucleotide polymorphism (SNP), insertion (INS), and deletion (DEL) under different expression levels of APOBEC3B, based on the WES data from TCGA portal in which the mutations had been called by VarScan2. As shown in **Figure 5A**, most genomic variants were nonsense mutation, missense mutation, and silent in the APOBEC3B^high^ and APOBEC3B^low^ groups. As for SNVs, the mutation numbers of T>A, C>T, C>G, and C>A in APOBEC3B^high^ cohort were significantly higher than those in APOBEC3B^low^ cohort (**Figure 5B**). Furthermore, SNPs in the APOBEC3B^low^ cohort were outnumbered by those in the APOBEC3B^high^ cohort; however, INS and DEL in two cohorts showed no significant difference (**Figure 5C**). Moreover, the mutation frequencies of some genes differed from these two groups, and the top 10 mutated genes were exhibited in **Figure 5D**. Common carcinogenic pathways were found to be more active in APOBEC3B^high^ group (**Figures 5E, G**). The strongest co-occurrent pairs of gene alteration in the APOBEC3B^high^ group were ATRX-TP53, and in the APOBEC3B^low^ groups were ATRX-TP53 as well as ATRX-IDH1, which was in line with previous studies> (19–21) Meanwhile, the most mutually exclusive pairs in APOBEC3B^high^ and APOBEC3B^low^ groups were CIC-TP53 and EGFR-IDH1, respectively (**Figures 5F, H**).

### Genes Positively Related to APOBEC3B Are Enriched in Immune and Inflammatory Related Pathways

**Figure 6:**
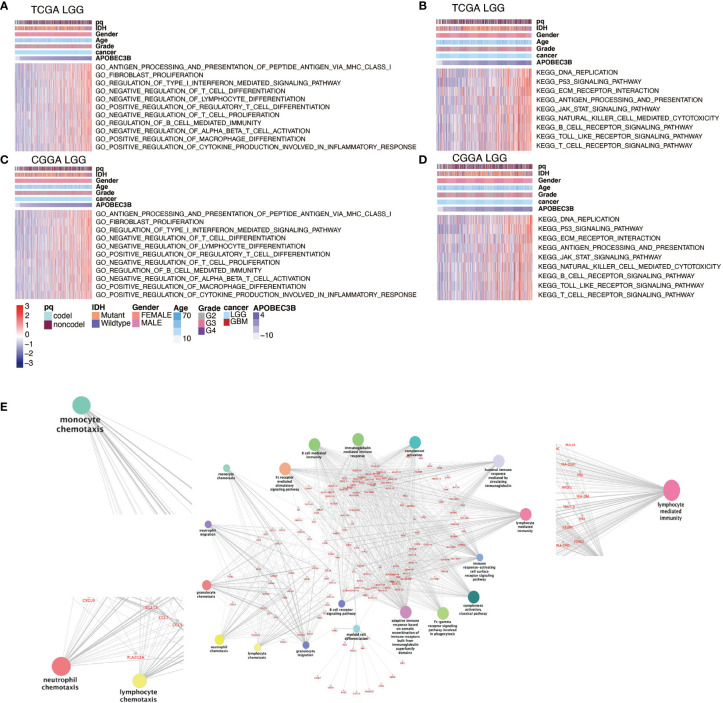
APOBEC3B-related biological functions in LGG. GO analysis based on APOBEC3B levels in **(A)** TCGA and **(C)** CGGA datasets in LGG patients. KEGG pathway analysis based on APOBEC3B expression levels in **(B)** TCGA and **(D)** CGGA datasets in LGG patients. **(E)** The APOBEC3B related pathway revealed by APOBEC3B positively associated genes in TCGA datasets with ClueGO.

We using GO analysis and KEGG pathway analysis to further investigate the potential function of APOBEC3B in the development of human glioma. Our results revealed that several immune and inflammatory related pathways were involved in APOBEC3B-mediated immune microenvironment. GO results revealed APOBEC3B was significantly correlated with type 1 interferon, MHC-I and cytokine-mediated signaling pathway, negatively regulates differentiation of T cell, positively regulates regulatory T cell and macrophage as well as fibroblast proliferation in LGG (**Figures 6A, C**) and pan-glioma analysis (**Figures S3A, C**) from TCGA and CGGA. The signaling network from KEGG pathway analysis further elucidates the relevance between APOBEC3B and immune and inflammation related pathways including antigen processing and presentation, p53, JAK-STAT and T, B cell receptor signaling pathway in LGG patients (**Figures 6B, D**) and pan-glioma analysis (**Figures S3B, D**). As shown in **Figure 6E**, the result of GO pathway analysis revealed that APOBEC3B was significantly related to immune infiltrating, such as monocyte chemotaxis, neutrophil chemotaxis, lymphocyte chemotaxis, and lymphocyte mediated pathways. These data suggest APOBEC3B might play an immunosuppression role in the TME of glioma.

### APOBEC3B Is Correlated With Inflammatory Activities in Gliomas

A positive feedback loop of APOBEC3B and inflammatory response mediator IL-6 has been found in hepatocellular carcinoma through the JAK1/STAT3 pathway (22). Meanwhile, based on our analysis, APOBEC3B was also involved in inflammatory responses in glioma. We further observed that APOBEC3B was positively correlated with MHC-1, MHC-2, STAT1, IFN, LCK, and HCK metagenes, but negatively related to IgG metagene, a marker for B cells in LGG patients (**Figures S4A, B**) and pan-glioma analysis (**Figures S4C, D**) in TCGA and CGGA datasets.

### APOBEC3B Is Related to Immune and Stromal Cell Infiltration in Gliomas

**Figure 7:**
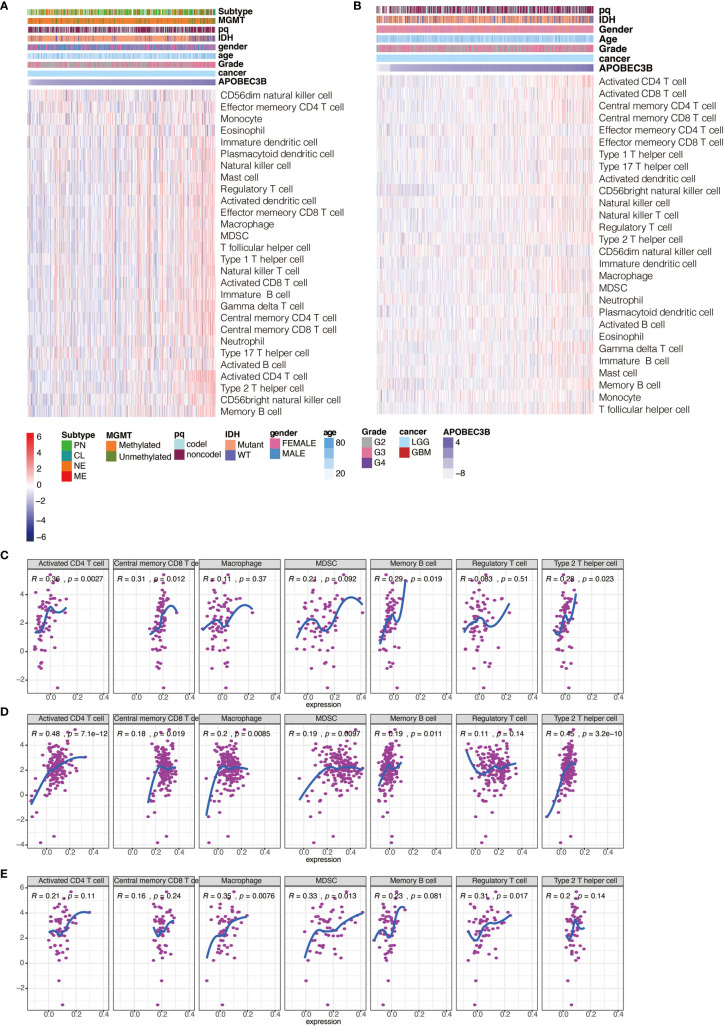
Heatmaps illustrating the relationship between APOBEC3B and immune cell populations based on **(A)** TCGA and **(B)** CGGA in LGG patients. Correlation of APOBEC3B expression with immune infiltration cells including activated CD4+T cells, central memory CD8+T cells, macrophages, MDSC, memory B cell, regulatory T cells and Th2 cells in **(C)** KICH, **(D)** PAAD< **(E)** UCS.

We further explore the relevance of APOBEC3B expression and ESTIMATE scores. Our results illuminated that APOBEC3B expression was positively related to the immune score, stromal score, and estimate score in the pan-glioma analysis (**Figure S5A**) and LGG (**Figure S5B**) respectively. Immune suppression is a significant feature of human gliomas which partly ascribes to TME components. To further understand in-depth the relevance between elevated APOBEC3B and immune tumor microenvironment, we examined which immune-related cell types are influenced by APOBEC3B in glioma. Using cell type enrichment analysis, we observed that APOBEC3B was strongly positively correlated with activated CD4^+^ T cell, *γδ* T cell, NK cells, dendritic cells and myeloid-derived suppressor cells in LGG patients (**Figures 7A, B**) and pan-glioma analysis (**Figures S6A, B**). Moreover, specific stromal cell types like fibroblasts, epithelial cells, and monocyte are related to glioma in LGG patients (**Figures S5C, D**) and pan-glioma analysis as well (**Figures S6C, D**). Taken together, our results suggested that increased APOBEC3B tend to recruit immune and stromal cells into the tumor microenvironment in glioma.

### Correlation Between APOBEC3B and Immune Cells in Pan-Cancer

To further understand the relationships between APOBEC3B and infiltrating immune cells in TME, we analyzed the correlation between APOBEC3B and several immune cells such as activated CD4^+^ T cells, central memory CD8^+^ T cells, macrophages, MDSCs, memory B cells, regulatory T cells, and type 2 T helper cells in pan-cancer. And we found in KICH (**Figure 7C**), PAAD (**Figure 7D**) and UCS (**Figure 7E**), APOBEC3B expression was positively correlated with these immune cells. In accordance with the results in gliomas, APOBEC3B expression in UCS was mostly correlated with macrophage, MDSC, regulatory T cell and type 2 helper cell, which contributed to immunosuppression in TME, but these immunosuppressive cells are less significantly correlated with APOBEC3B in KICH and PAAD.

### APOBEC3B Is Correlated With Other Immune Checkpoint Molecules in Gliomas

**Figure 8:**
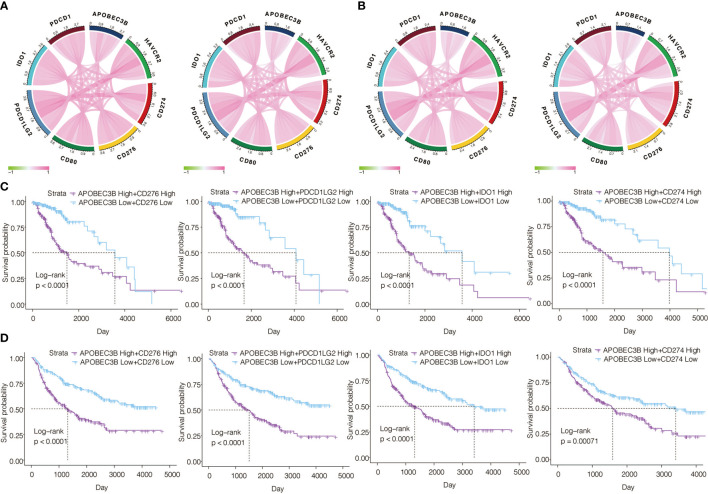
Correlation of APOBEC3B expression with other immune checkpoint molecules in gliomas. Correlation analyses of APOBEC3B and other immune checkpoints in **(A)** pan-glioma analysis and **(B)** LGG patients from TCGA (left) and CGGA (right) datasets. Analyzing combined prognostic value of APOBEC3B and CD276, PDCD1LG2, IDO1 and CD274 expression in LGG patients from **(C)** TCGA and **(D)** CGGA datasets.

Regarded as a prospective immunotherapy, immune checkpoint inhibitors take an important role in the regulation of immune response in cancers. We investigated the relationship between APOBEC3B and several immune checkpoint genes in gliomas. We found APOBEC3B was associated with CD276(B7-H3), PDCD1LG2(PD-L2), IDO1, CD274(PD-L1), HAVCR2(TIM-3), and CD80(B7-1) in pan-glioma analysis (**Figure 8A**) and LGG (**Figure 8B**) in TCGA and CGGA datasets. We further analyzed the prognostic value of APOBEC3B in combination with CD276, PDCD1LG2, IDO1, and CD274. Significant worse prognosis was observed in the patient group with the co-upregulation of APOBEC3B and these genes in LGG patients from TCGA and CGGA databases (**Figures 8C, D**).

### Identification of a Gene Signature Associated With Immune Cells

**Figure 9:**
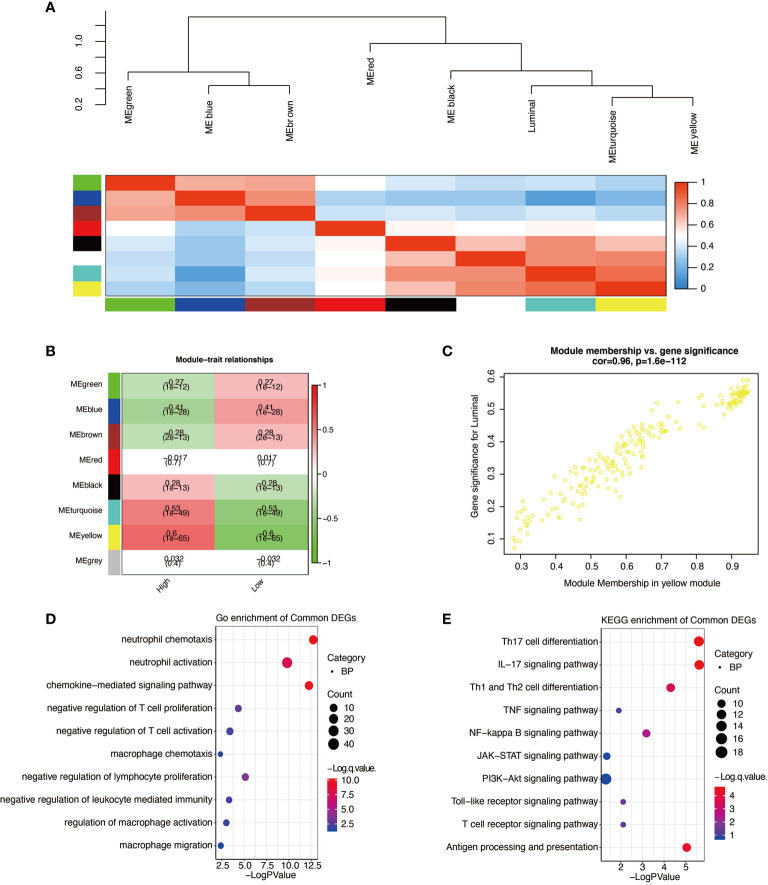
Higher expression of APOBEC3B related gene signature identification. **(A)** WGCNA was applied to identify the clustered eigengene modules. **(B)** Seven modules are identified by WGCNA. **(C)** The yellow module has the highest correlation (r = 0.96, P = 1.6e-112). **(D)** Go analysis was performed based on APOBEC3B^high^ related genes. **(E)** KEGG analysis was performed based on APOBEC3B ^high^ related genes.

WGCNAs were applied to determine the genes most correlated with high expression of APOBEC3B. Genes were clustered into eight modules (**Figures 9A**), and the correlation between the eight modules and the expression level of APOBEC3B was shown in **Figure 9B**. The yellow module showed the highest correlation coefficient with high APOBEC3B expression level. A significant correlation between module membership in the yellow module and gene significance for APOBEC3B^high^ was observed (**Figure 9C**). Then, GO enrichment analysis revealed that the neutrophil activation, neutrophil chemotaxis, and chemokine-mediated signaling pathway were the most related gene functions associated with the high expression of APOBEC3B (**Figure 9D**). And KEGG enrichment analysis revealed that Th17 cell differentiation, IL-17 signaling pathway, antigen processing and presentation, Th1 and Th2 cell differentiation as well as NF-kappa B signaling pathway were the most related pathways involved in high expression of APOBEC3B (**Figure 9E**).

## Discussion

Somatic mutations are responsible for the transformation from normal cells to cancer cells. Generally, somatic mutation has been considered as a therapy evasion promoter of cancer. Correspondingly, mutation can also promote antitumor T-cell response. As the only member of the deaminase family with constitutive nuclear localization, APOBEC3B, the endogenous mutagenic factor, can induce genomic C-to-U lesions that are correlated with a variety of mutagenic outcomes (23). Therefore, we are interested in the characteristics of APOBEC3B in the development of glioma.

To the best of our knowledge, studies about expression and prognostic value of APOBEC3B have been conducted in several cancer types. For example, highly expressed APOBEC3B is regarded as an unfavorable prognostic factors in myeloma (11), ovarian cancer (24), and clear cell renal cell carcinoma (25). Immune-oncology has become a hot area for tumor therapy nowadays. However, the immune-related and mutation-related role of APOBEC3B in cancer metastasis has not been thoroughly investigated. Most recently, the duality of APOBEC3B in immunotherapy has been demonstrated, in which APOBEC3B not only acts as the general driving force of therapy escape but also significantly activates the immune system in melanoma (15). As important parts of TME, inflammatory cells and infiltrating immune cells are closely related to curative effect. Thus, understanding the TME can help to unveil the mechanisms of tumor development and shed light on tumor therapy. Previous study has proved that APOBEC3B is related to an active immune infiltration in high-grade serous ovarian carcinoma (26). But the activation of tumor-infiltrating immune cells also mediates APOBEC3B deletion in breast cancer in Asian patients (27).

In the current study, we characterized the landscape of APOBEC3B among glioma and other cancers *via* a large-scale bioinformatic analysis. We observed that APOBEC3B expression was upregulated in numerous cancer categories. In gliomas, the increasing expression level of APOBEC3B is consistent with the increasing grade of gliomas based on WHO classification. Presumable worse prognosis was observed in glioma patients with higher expression of APOBEC3B, and the result was further verified in ACC, CHOL, ESCA, LIHC, LUAD, PAAD, UCEC, UCS, and KICH in our study. Meanwhile, APOBEC3B was closely related to oncogenic mutation in our study, indicating its role in carcinogenesis. Thus, APOBEC3B has prognostic value in pan-cancer.

In GO and KEGG analysis, another major finding of our study was that elevated APOBEC3B was significantly accompanied by inflammatory, stromal and immune related signaling pathways in LGG, among which fibroblast proliferation, negative regulation of T cell, positive regulation of Tregs and cytokines productions were most significant. Furthermore, upregulated APOBEC3B was significantly associated with immune cells and stromal cells’ infiltration in glioma based on ESTIMATE algorithm. Several immune infiltrating cell types possess the features of immunosuppression: It is well documented that MDSC is able to inhibit innate and adaptive immunity (28), and macrophages have been indicated to promote cancer cell proliferation, immunosuppression, and angiogenesis in cancers (29). Treg not only can suppress the activation and expansion of different effector cells from mediating autoimmunity, but also can negatively affect immune therapies concerning immune checkpoints inhibitors (30, 31). Moreover, Th2 responses are generally considered undesirable since they mitigate against cytotoxic antitumor immune mechanisms in glioma (31). Cell type enrichment analysis further revealed that APOBEC3B was significantly correlated with MDSC, macrophage, regulatory T cells, and Th2 cells in glioma, KICH, PAAD, and UCS, providing evidence to the statement that APOBEC3B was an immunotherapy escape driver in LGG. Taken together, we proposed that APOBEC3B may be involved in the regulation of immunosuppressive microenvironment by recruiting immunosuppression cells and might become a selective target to inhibit immunosuppression.

The efficient treatment option for glioma is limited. Diverse cancer immunotherapeutic approaches have exhibited significant and exciting treatment outcomes for several cancer types, and have also triggered unparalleled research interest in glioma. Nowadays, glioma immunotherapy research predominantly focuses on immunosuppressive ICBs, CAR-T cells, vaccine, and oncolytic viruses (7). Although blood brain barrier and immunosuppressive TME in glioma patients suppress the efficiency of ICB treatment, ICBs do have revolutionized the treatment of solid malignant tumor. Our analysis illuminated that APOBEC3B was correlated with immune checkpoints including CD276, PDCD1LG2, IDO1, CD274, and TIM-3 in LGG and pan-glioma analysis. These immune checkpoints are promising immunotherapeutic targets for glioma, which CD276, PDCD1LG2, IDO1 and TIM-3 are both unfavorable prognosticator for glioma patients (32–35). In our current study, the co-upregulation of APOBEC3B and CD276, PDCD1LG2, IDO1, and CD274 suggested worse survival probability. CD276 has become a novel Cart-T target for GBM (36) while inhibition of PD-1/PD-L1 pathway can be a latent treatment strategy for glioma (37). Other promising immune checkpoint molecules like 4-1BB, GITR, and TIGIT are further being considered to enter early phase clinical trials (38). Immune checkpoints also take part in immunosuppression: upregulating PD-L1 can bind receptors on immune cells and suppress lymphocyte activation (39, 40). The correlation between APOBEC3B and these classic immune checkpoint molecules indicates that targeting APOBEC3B may become a potential approach for mediating immunotherapeutic response in LGG patients.

WGCNA is well applied to classify the high-throughput sequencing data into subsets of genes with cell-specific expression; therefore, we applied WGCNA to identify the APOBEC3B^high^ related genes. In our study, the yellow module was the most correlated one; further GO analysis revealed that neutrophil activities and chemokine-mediated signaling pathway were the most represented activities related to higher expression of APOBEC3B. Moreover, negative regulation of T cell activities further demonstrated that higher expression of APOBEC3B was correlated with activated inflammation and immunosuppression. In the KEGG analysis, IL-17 signaling pathway was the most relevant activity that occurred in patients with higher level of APOBEC3B. IL-17 is a cytokine produced by Th17 cell, suggesting the crucial role of Th17 cell in glioma pathogenesis.

In summary, our results revealed that APOBEC3B overexpression was related to aggressive clinicopathologic features, poor prognosis, inflammatory and immune pathways in glioma. These findings may be helpful in further optimizing diagnosis and immune treatments for LGG.

## Data Availability Statement

All data used in this work can be acquired from the Cancer Genome Atlas (TCGA) datasets (https://xenabrowser.net/), the Chinese Glioma Genome Atlas (CGGA) datasets (http://www.cgga.org.cn/).

## Author Contributions

HZ and QC conceptualized and designed the study. QC and ZL provided foundation support. HZ and ZC acquired and analyzed the data. HZ and ZC interpreted the data. HZ, ZC, ZW, ZD, ZH, XZ and QC drafted the manuscript and revised it for submission quality. All authors contributed to the article and approved the submitted version. QC supervised the study.

## Funding

This work was supported by the National Natural Science Foundation of China (Nos. 82073893, 81703622, 81472693, and 81873635), China Postdoctoral Science Foundation (No. 2018M633002), Hunan Provincial Natural Science Foundation of China (No. 2018JJ3838), Hunan Provincial Health and Health Committee Foundation of China (C2019186).

## Conflict of Interest

The authors declare that the research was conducted in the absence of any commercial or financial relationships that could be construed as a potential conflict of interest.

## Supplementary Material

Supplementary Figure 1 Relationship between APOBEC3B and methylation statues. **(A).** Relationship between APOBEC3B and the mean value of methylation status at promoter region in TCGA. Relationship between APOBEC3B and methylation status at promoter region in TCGA: **(B)** cg01089751, **(C)** cg06837067, **(D)** cg11816043, **(E)** cg14194956, **(F)** cg14387414, **(G)** cg16045423, **(H)** cg21707131, **(I)** cg25787886, **(J)** cg26000393. The orange dots represent IDH-mutant samples, and cyan dots represent IDH wild-type samples, respectively. The orange line and cyan line represent linear regression between APOBEC3B expression and promoter region methylation in IDH-mutant samples and IDH wild-type samples, respectively.

Supplementary Figure 2 Relationship between APOBEC3B and **(A)** anatomic structure analysis. CT (Cellular Tumour), HBV (Hyperplastic Blood Vessels), IT (Infiltrating Tumour), LE (Leading Edge), MVP (Microvascular Proliferation), PAN (Pseudopalisading Cells Around Necrosis) and PNZ (Perinecrotic Zone). **(B)** APOBEC3B copy number in TCGA pan-glioma. **(C)** distinct radiographical regions of glioma. **(D)** different histology analysis from CGGA database. **(E)** different disease conditions including primary, recurrent and secondary from CGGA database. **(F)** different treatment outcomes.

Supplementary Figure 3 APOBEC3B-related biological functions in gliomas. GO analysis based on APOBEC3B levels in **(A)** TCGA and **(C)** CGGA datasets in pan-glioma analysis. KEGG pathway analysis based on APOBEC3B expression levels in **(B)** TCGA and **(D)** CGGA datasets in pan-glioma analysis.

Supplementary Figure 4 Heatmaps illuminating APOBEC3B related inflammatory activities in LGG and pan-glioma. Analysis between APOBEC3B and inflammatory metagenes in LGG from **(A)** TCGA and **(B)** CGGA datasets and pan-glioma analysis from **(C)** TCGA and **(D)** CGGA.

Supplementary Figure 5 Correlation between APOBEC3B expression and ESTIMATE algorithm scores in gliomas. APOBEC3B expression was positively correlated with immune score, stromal score and ESTIMATE score in **(A)** pan-glioma analysis and **(B)** LGG patients. The relationship between APOBEC3B and stromal cell populations based on **(C)** TCGA and **(D)** CGGA in LGG patients.

Supplementary Figure 6 Heatmaps illustrating the relationship between APOBEC3B and immune cell populations based on **(A)** TCGA and **(B)** CGGA in pan-glioma analysis. The relationship between APOBEC3B and stromal cell populations based on **(C)** TCGA and **(D)** CGGA in pan-glioma analysis.
