# Supplementary figures and images for: Correlation Between APOBEC3B Expression and Clinical Characterization in Lower-Grade Gliomas
Source: Front Oncol. 2021 Mar 26;11:625838. doi: 10.3389/fonc.2021.625838 (PMC8033027; doi:10.3389/fonc.2021.625838)

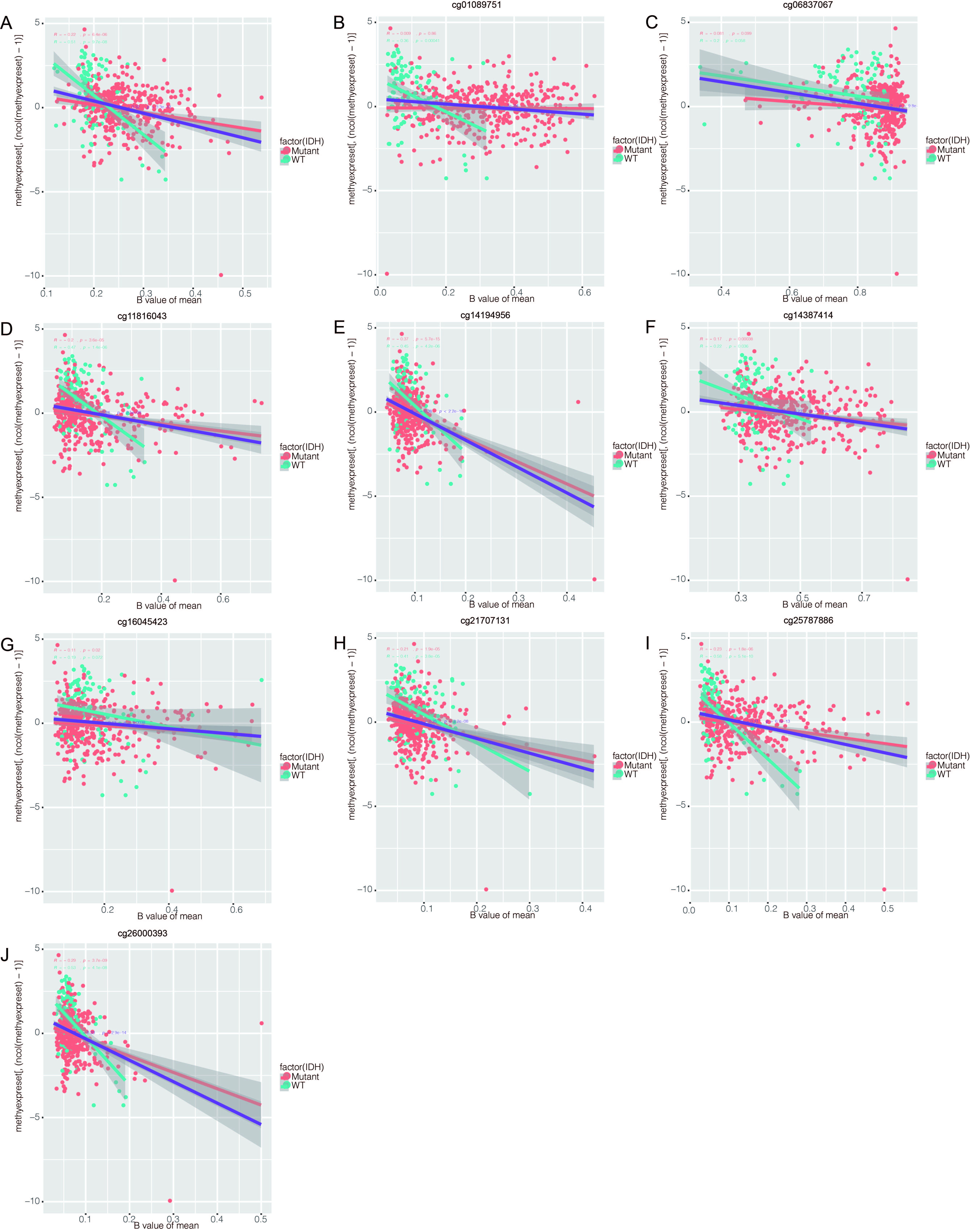

Supplement: Supplementary Figure 1 — Relationship between APOBEC3B and methylation statues. (A). Relationship between APOBEC3B and the mean value of methylation status at promoter region in TCGA. Relationship between APOBEC3B and methylation status at promoter region in TCGA: (B) cg01089751, (C) cg06837067, (D) cg11816043, (E) cg14194956, (F) cg14387414, (G) cg16045423, (H) cg21707131, (I) cg25787886, (J) cg26000393. The orange dots represent IDH-mutant samples, and cyan dots represent IDH wild-type samples, respectively. The orange line and cyan line represent linear regression between APOBEC3B expression and promoter region methylation in IDH-mutant samples and IDH wild-type samples, respectively. [file Image_1.jpeg]

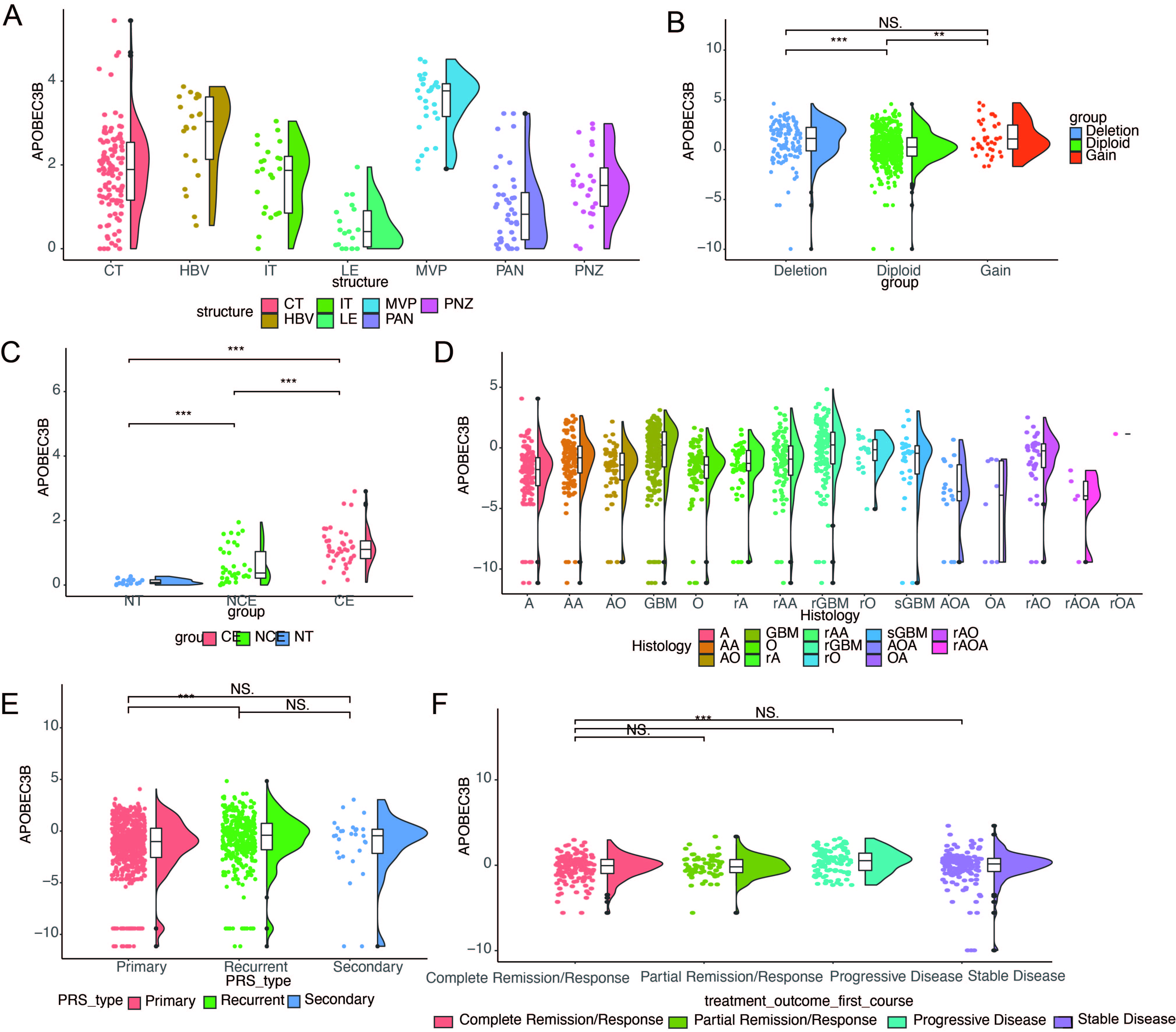

Supplement: Supplementary Figure 2 — Relationship between APOBEC3B and (A) anatomic structure analysis. CT (Cellular Tumour), HBV (Hyperplastic Blood Vessels), IT (Infiltrating Tumour), LE (Leading Edge), MVP (Microvascular Proliferation), PAN (Pseudopalisading Cells Around Necrosis) and PNZ (Perinecrotic Zone). (B) APOBEC3B copy number in TCGA pan-glioma. (C) distinct radiographical regions of glioma. (D) different histology analysis from CGGA database. (E) different disease conditions including primary, recurrent and secondary from CGGA database. (F) different treatment outcomes. [file Image_2.jpeg]

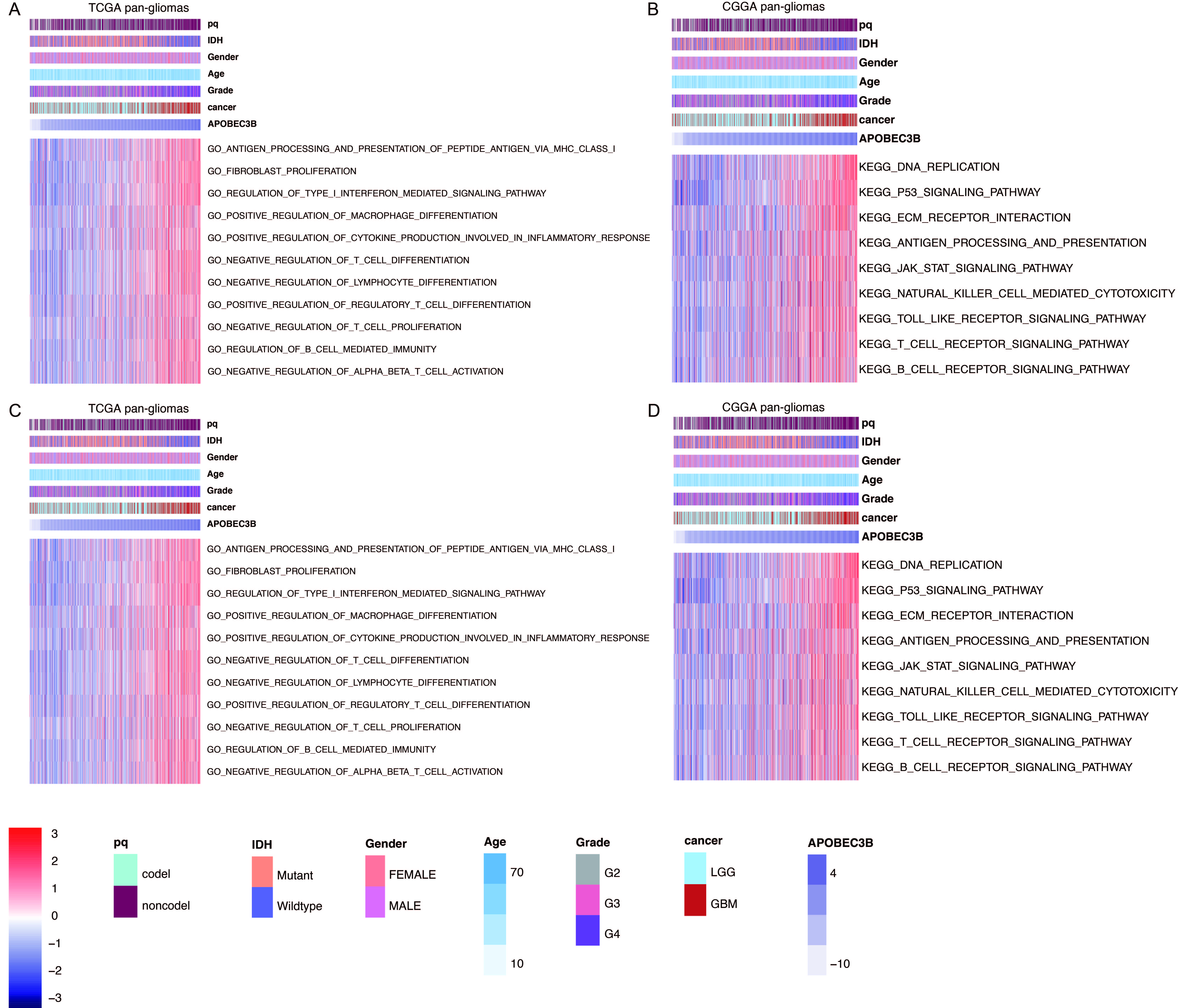

Supplement: Supplementary Figure 3 — APOBEC3B-related biological functions in gliomas. GO analysis based on APOBEC3B levels in (A) TCGA and (C) CGGA datasets in pan-glioma analysis. KEGG pathway analysis based on APOBEC3B expression levels in (B) TCGA and (D) CGGA datasets in pan-glioma analysis. [file Image_3.jpeg]

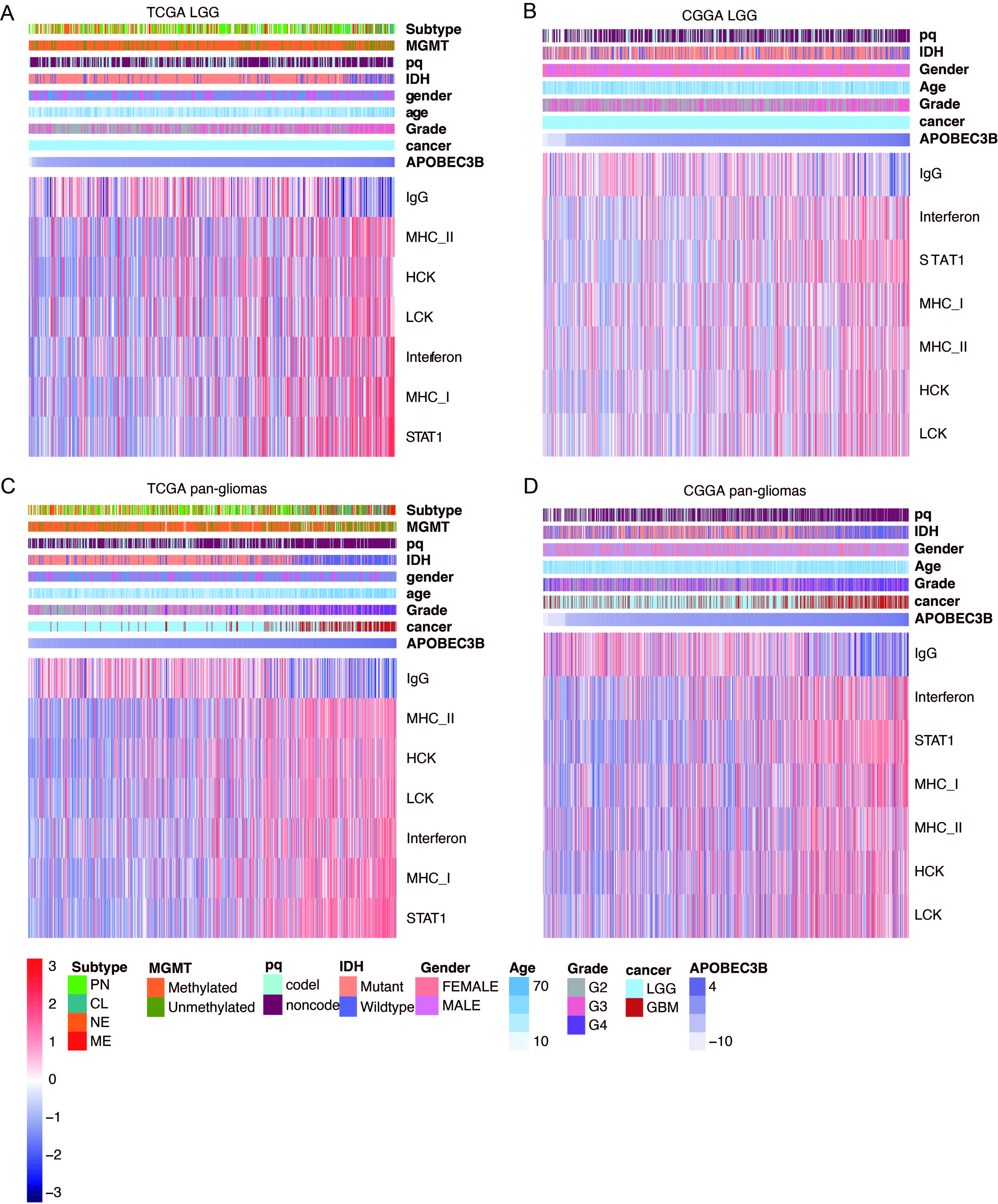

Supplement: Supplementary Figure 4 — Heatmaps illuminating APOBEC3B related inflammatory activities in LGG and pan-glioma. Analysis between APOBEC3B and inflammatory metagenes in LGG from (A) TCGA and (B) CGGA datasets and pan-glioma analysis from (C) TCGA and (D) CGGA. [file Image_4.jpeg]

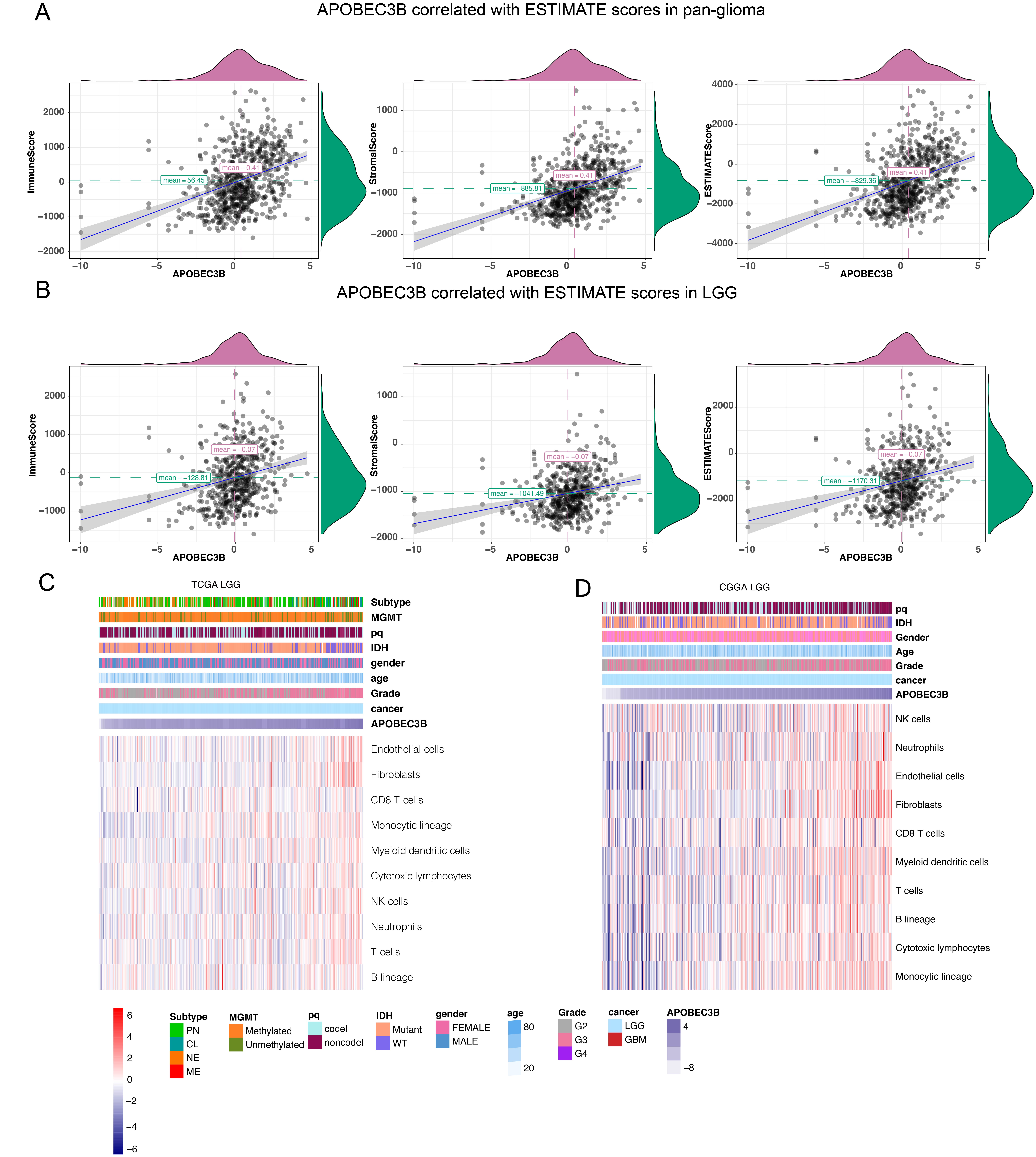

Supplement: Supplementary Figure 5 — Correlation between APOBEC3B expression and ESTIMATE algorithm scores in gliomas. APOBEC3B expression was positively correlated with immune score, stromal score and ESTIMATE score in (A) pan-glioma analysis and (B) LGG patients. The relationship between APOBEC3B and stromal cell populations based on (C) TCGA and (D) CGGA in LGG patients. [file Image_5.jpeg]

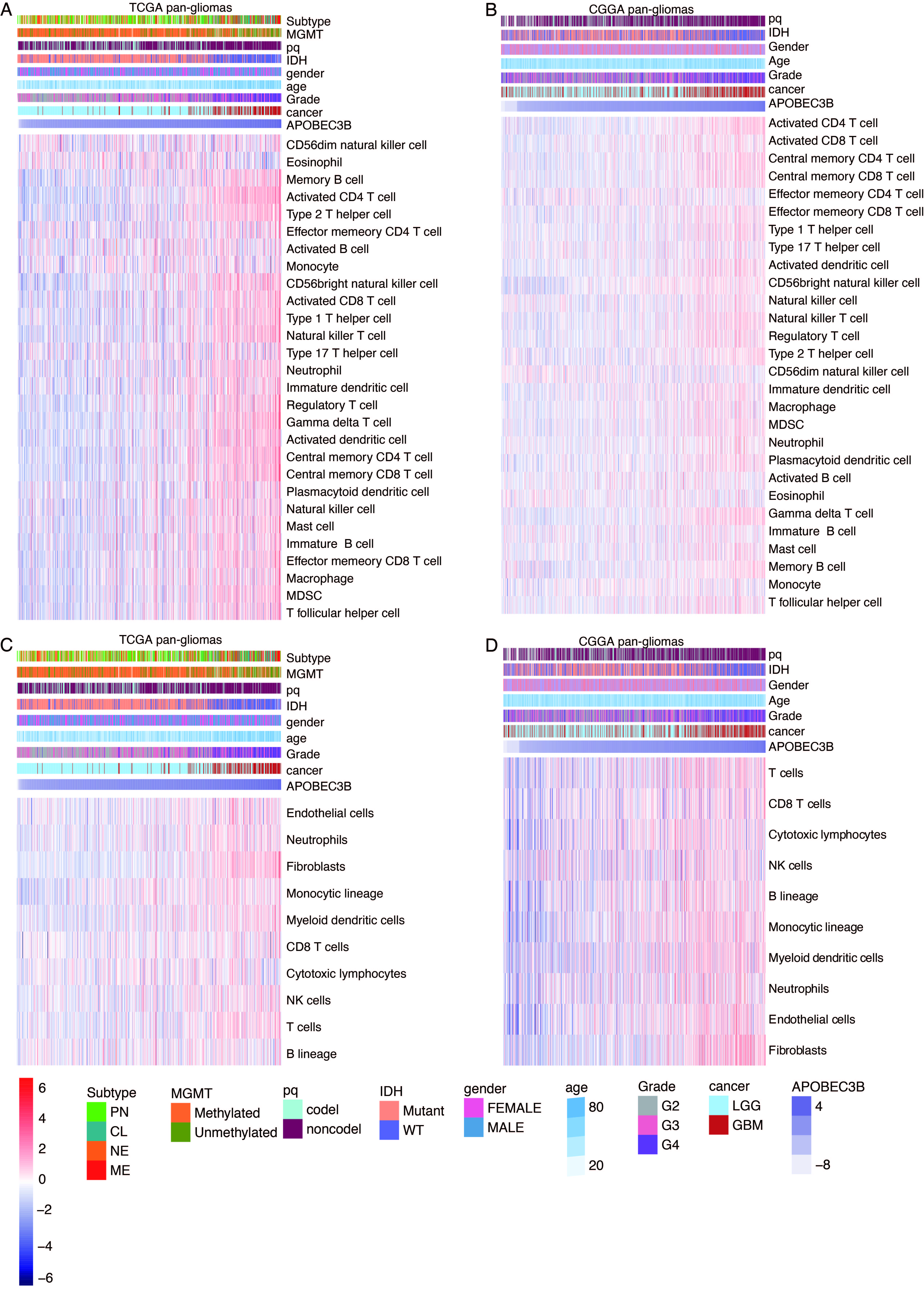

Supplement: Supplementary Figure 6 — Heatmaps illustrating the relationship between APOBEC3B and immune cell populations based on (A) TCGA and (B) CGGA in pan-glioma analysis. The relationship between APOBEC3B and stromal cell populations based on (C) TCGA and (D) CGGA in pan-glioma analysis. [file Image_6.jpeg]
